# Challenges and reforms in Spain’s health technology assessment system: analysis of criteria influencing medicines’ reimbursement decisions between 2019 and 2022 in Spain

**DOI:** 10.1007/s10198-025-01790-7

**Published:** 2025-05-09

**Authors:** Pilar Pinilla-Dominguez, Jaime Pinilla-Dominguez

**Affiliations:** 1 Department of Quantitative methods in Economics and Management, School of Economics, University of Las Palmas de Gran Canaria (ULPGC), Las Palmas, Spain; 2 NICE Advice, National Institute for Health and Care Excellence, London, UK

**Keywords:** Pricing and reimbursement, Health technology assessment, Pharmaceutical policy

## Abstract

**Objective:**

To analyse the criteria influencing medicines’ reimbursement decisions in the Spanish National Health Service and assess the extent to which these decisions have been supported by health technology assessment (HTA), considering the ongoing HTA reform.

**Materials and methods:**

The sample includes all new medicines and new indications undergoing reimbursement between May 2019 and December 2022 in Spain. Criteria influencing the decision were derived from the Interministerial Pricing Committee for Medicines’ reports. These were matched with the HTA reports obtained from the Spanish Medicines Agency’s website. Spanish decisions are compared to those in France and England. The analyses include descriptive analysis, association statistical tests, sentiment text analysis, keyword extraction, decision analysis, and clustering.

**Results:**

Out of 477 therapeutic indications, 253 could be matched to a HTA report. Positive recommendations (*n* = 110) were statistically significantly associated with severity and therapeutic value (including clinical and cost effectiveness) criteria, whereas negative recommendations (*n* = 143) were mostly associated with criteria based on budget impact and availability of a cheaper alternative option (*p* < 0.05). The innovation criterion was not used to support any decision. Only 9.49% of reimbursement reports mentioned the HTA in the conclusions, and 21.74% of the HTAs included keywords aligned with the specific decision-making criteria.

**Conclusion:**

The criteria used to justify the reimbursement decisions of medicines in Spain do not align with the information included in the HTA. This discrepancy highlights the need for the ongoing HTA reform to develop an appraisal framework that aligns with the HTA assessment in a transparent, rigorous, and inclusive manner.

**Supplementary Information:**

The online version contains supplementary material available at 10.1007/s10198-025-01790-7.

## Introduction

Health technology assessment (HTA) is considered an evidence-based tool that supports and informs decision making on the pricing and reimbursement of health technologies [1]. However, despite the existence of established HTA processes in many countries, their impact in actually supporting and informing final decision making is unclear [2].

In August 2024, the Ministry of Health in Spain published a draft of the new royal decree regulating the evaluation of health technologies including drugs, medical procedures, medical devices, in vitro diagnostic tests and other health-related technologies [3]. This draft royal decree follows a court ruling decision published in 2023 by the Spanish court, which declared void and null the Spanish medicines evaluation process based on the so-called therapeutic positioning report (in Spanish: informe de posicionamiento terapuetico [IPT]) [4]. This draft royal decree on the evaluation of health technologies was followed by the publication of the new Spanish strategy of the pharmaceutical industry and the project of an additional new royal decree which regulates the pricing and reimbursement system for medicines, both published at the end of 2024. These documents showcase the depth and scope of the ongoing reform of the evaluation and pricing and reimbursement system for medicines in Spain [3, 5, 6].

Until 2023, the IPT was meant to be the key resource informing the final reimbursement decision by the Spanish Ministry of Health. This decision also had to be justified on the basis of the criteria specified in the legislation, which include (a) severity; (b) specific needs of certain subgroups; (c) social and therapeutic value of the medicines and incremental clinical benefit considering also its cost effectiveness; (d) rationing of public spending on medicines and budget impact from the perspective of the Spanish National Health Service (SNHS); (e) availability of alternative therapeutic options for the condition at the same or inferior price to that of the medicine under consideration; and (f) innovation (see Box S1 in the supplementary material) [7, 8]. The assessment, appraisal and pricing and reimbursement decision steps and the personnel involved in these tasks were not clearly delineated and differentiated. Despite different procedural reforms, the quality of the IPTs was questionable and their usability for supporting pricing and reimbursement decisions of medicines was unclear, with several authors highlighting the lack of transparency, governance and methodological best practice of the Spanish HTA and pricing and reimbursement process for medicines [9–12]. This was even further explored by independent experts and advisors to the Ministry of health who suggested concrete steps to improve the process [13].

The ongoing reform envisages the separation of functions for the assessment, therapeutic positioning of the medicine and final decision making [3]. Specifically, the creation of an “Office for the evaluation of the efficiency of health technologies” will focus on the assessment of the evidence, while a separate “Group for the positioning of health technologies” will do a final appraisal based on the HTA assessment and other relevant evidence, and will also derive a recommendation on the appropriate therapeutic positioning of the medicine. The pharmaceutical industry strategy and the project of the new royal decree for regulating the pricing and reimbursement system for medicines both commit to the revision and further definition of the criteria used for final pricing and reimbursement decisions of medicines, to be linked to the HTA process, although specific details are yet to be provided. All these legislative documents are meant to be complemented with several methodological guidelines and processes, such as the recently published guide for the economic evaluation of medicines [14].

This reform arrives at a time when change is nevertheless required in line with the implementation of the EU HTA legislation 2021/2282 in Europe in 2025, which includes the development of joint clinical assessments and their mandatory consideration by member states [15–17]. Starting from 2025, new oncology and advanced therapy medicinal products will be clinically assessed at the EU level, with orphan medicines and all new medicines following suit in subsequent years. The legislation allows each country to conduct further assessments as long as these are not a duplication of the joint ones. These further assessments can include other criteria such as economic or ethical. The appraisal and subsequent decision-making will also be responsibility of the individual countries. The challenge lies in ensuring that these assessments remain relevant and are meaningfully integrated into the national decision-making process and complemented with other evidence required for decision-making. All the legislative documents covering the ongoing reform in Spain reference the alignment of the new process to the EU HTA legislation, and the need to expand it to describe and define areas not covered by the joint clinical assessments, such as the categorisation of the added clinical benefit or economic aspects including economic evaluation and budget impact. Table S1 in the supplementary material compares key aspects of the HTA and pricing and reimbursement process for medicines in Spain and the new proposals within the ongoing reform, and the alignment with the EU HTA regulation.

The objective of this study is to showcase critical issues within the Spanish HTA system that need urgent revision within the current Spanish HTA reform. Proposing a new regulation without studying in detail the various interactions and connections of the current decision-making system could lead the Spanish Ministry of Health to inadvertently retain inefficiencies in the HTA and pricing and reimbursement processes. To highlights the relevance of these critical interactions, we conducted an in-depth analysis of the criteria that have influenced the decisions on pricing and reimbursement of medicines in the SNHS and investigated whether and how these have been supported by the corresponding HTA assessments based on the IPTs. The study also aims to compare the Spanish decisions with those made by HTA agencies in other countries such as the French National Authority (in French: Haute Autorité de Santé, [HAS]) in France and the National Institute for Health and Care Excellence (NICE) in England. Spain is suggesting the categorisation of the added clinical benefit of the medicine in the new system, and France has an established process to conclude on the added therapeutic benefit [18]. Spain is also aiming to expand and formally implement economic evaluations as part of the decision-making process and NICE in England is well-known for concluding on the clinical and cost-effectiveness of a medicine using a common outcome measure, the quality-adjusted life year (QALY) as part of their HTA [19].

The methodology in this study includes descriptive analysis, association statistical tests, sentiment text analysis, keyword extraction, decision analysis, and clustering to evaluate and interpret the IPT conclusions. This approach ensures a standardised, data-driven method for assessing the IPTs and the final reimbursement decision.

The results and conclusions of this study lead to policy considerations that aim to support the current Spanish HTA reform including how the criteria considered for decision-making should align with the evidence available from the assessments. The analysis could also be of use to other countries facing a similar situation.

## Materials and methods

### Data collection

For our analysis, we identified all new medicines and new indications undergoing pricing and reimbursement between May 2019 (when the Ministry of Health started publishing details on the pricing and reimbursement decisions and the criteria used to base such decisions) and December 2022 (data cut-off for our analyses) in Spain. We focused on those medicines authorised by the centralised procedure in Europe. We downloaded all the available reports describing the pricing and reimbursement decision of the Interministerial Pricing Committee for Medicines (in Spanish: Comision interministerial de precios de medicamentos, CIPM) from the Ministry of Health’s website, conducted data extraction and developed a database [20]. We extracted the pricing and reimbursement decision, the criteria used to base this decision according to the criteria specified in the legislation. We double checked and completed this information with details from the Ministry of Health’s BIFIMED database, which includes information on the funding status of authorised medicines in Spain [21]. As we wanted to understand how the HTA assessments align with and support decision-making, we matched the dataset from the CIPM with the IPTs available for the medicines under analysis from the Spanish Agency of Medicines and Medical Products’ website [22]. We downloaded the IPTs and extracted detailed conclusions on the drug’s efficacy, safety, and potential incorporation into the SNHS including information on the assessments and relationship with the criteria used for decision-making (for example, if the IPT includes or mentions an economic evaluation, QALYs, or a budget impact analysis). The data were collected from official sources and compiled into an Excel file, with each row representing a unique therapeutic indication.

For the international comparison we extracted information from the NICE’s website on the appraisal of the medicines and the type of recommendation, and from the French government dataset on the HAS recommendation and conclusion on the level of added therapeutic value based on the ASMR scale, where I represents major therapeutic value and V represents no clinical improvement [23, 24].

### Descriptive analysis

As a first approximation to the content of our database we developed a frequency distribution analysis. To do this we created frequency tables to show the number or proportion of occurrences for each category for categorical variables in our study. We also use the sankey plot for the visualization of flow analysis between different states and variables. The sankey plot emphasises the major flows of the criteria used for the reimbursement decision.

### Data analysis

We conducted an analysis of qualitative variables for each indication based on the criteria used for decision-making and the corresponding funding decision. To explore the association between qualitative variables, we used the Fisher’s exact test. This test is useful to examine the significance of the association between two types of classification when sample sizes are small. The null hypothesis is that there is no association between the two variables. The alternative hypothesis is that there is an association between the two variables in any direction. A two- tailed p value < 0.05 was considered to reject the null hypothesis. In addition, we used a Cramér’s V correlation statistic as a measure of association between two nominal variables, which ranges from 0 to 1, where 0 indicates no relationship, and 1 represents a perfect relationship. A value of 0.2 or less indicates a weak relationship, while a value between 0.2 and 0.3 suggests a moderate relationship. All statistical analyses were performed using a standard software package (R, version. 4.3.3) [25].

### Sentiment score computation

We preprocessed the text data from the IPT’s conclusions to ensure consistency and accuracy in the subsequent analysis. The preprocessing steps included conversion to lowercase; punctuation removal; number removal; whitespace removal; and stopword removal. Using the ‘sentimentr’ package in R, we conducted sentiment analysis to quantify the overall sentiment expressed in each IPT’s conclusion [26].

First, we extracted the sentiment keywords, keywords that contributed positively or negatively to the sentiment of the text. A comprehensive list of these keywords and their frequency of occurrence can be found in the supplementary material, see Figure S1 and the file keywords.csv.

A sentiment score was calculated for each text segment, where the above keywords were identified. Linguistic modifiers such as negators, amplifiers, and adversative conjunctions that alter or intensify the meaning of the keywords were considered to refine sentiment score. A list of 140 potential modifiers can be found in Table S2 in the supplementary material. The sentiment scores were calculated using an algorithm that considers not only the polarity of keywords but also their context. This ensures a more accurate interpretation of text compared to simple word-based approaches. For a detailed explanation of the algorithm, refer to the sentimentr reference manual [27]. Finally, on the basis of the textual analysis, we categorised each drug as “recommended” or “not recommended” following the presence of positive keywords and the sentiment score.

### Linking to criteria, clustering and similarity analysis

We analysed each IPT’s conclusion in order to identify keywords relevant to the decision-making criteria specified in the CIPM. To evaluate the structural similarity of the IPT’s conclusions, a text similarity analysis was conducted. The text data were transformed into a term frequency matrix, and cosine distance was used to measure similarity. Cosine similarity is calculated by taking the dot product of each two text vectors and dividing it by the product of their magnitudes. Since cosine similarity ranges from 0 (completely dissimilar) to 1 (identical text), the cosine distance is equal to 1 − cosine similarity. This ensures that a higher cosine distance value means less similarity between the two IPT’s conclusion. We performed hierarchical clustering and Principal Component Analysis (PCA) to visualise and interpret the clustering results.

## Results

Between May 2019 and December 2022, there were 35 CIPM’s reports available including information on 477 therapeutic indications, out of which 253 could be matched to a published IPT. Over 90% of these indications were hospital drugs and almost 50% of them were anti-cancer drugs. The full therapeutic indications distribution by Anatomical Therapeutic Chemical (ATC) Classification System can be found in Table S3 in the supplementary material.

**Fig. 1:**
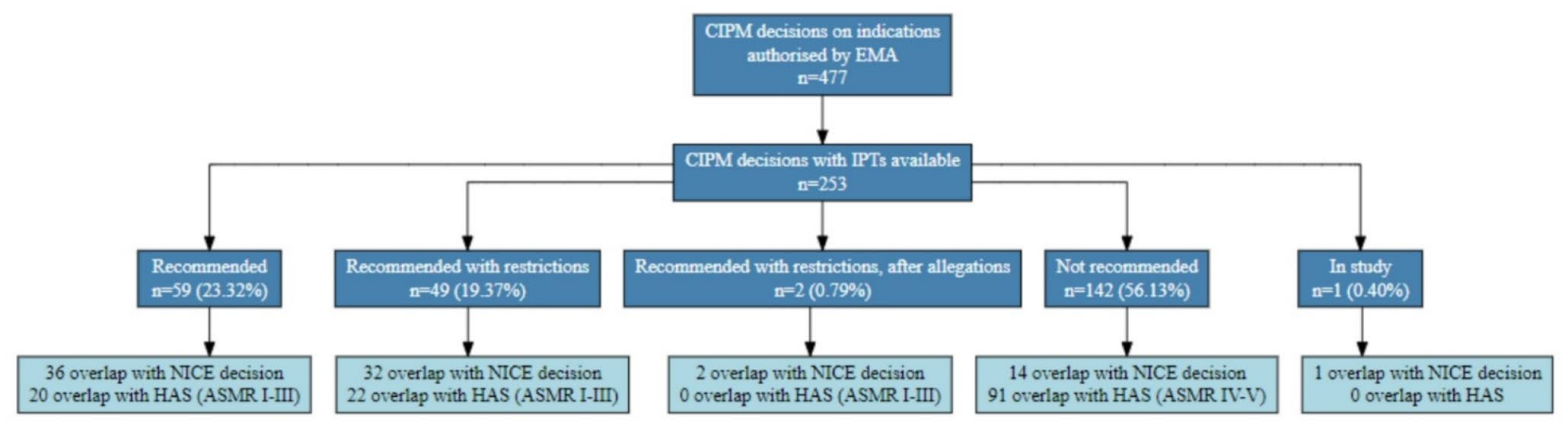
Reimbursement decisions of the Interministerial Pricing Committee on Medicines (CIPM) of indications that could be matched with a therapeutic positioning report (IPT) between May 2019 and December 2022 in the Spanish National Health Service, and similarities with France (HAS) and England (NICE) Abbreviations: ASMR: added therapeutic clinical benefit, CIPM: Interministerial Pricing Committee of Medicines; EMA: European Medicines Agency, HAS: Haute Autorité de santé, IPT: Therapeutic Positioning Report, NICE: National Institute for Health and Care Excellence

Out of the 253 indications, 59 (23.32%) were recommended for reimbursement, 1 (0.40%) was under study, 49 (19.37%) were recommended for reimbursement with some restriction in the indication, 2 (0.79%) were recommended for reimbursement after allegations with some restriction in the indication, and 142 (56.13%) were not recommended for reimbursement (see Fig. 1). The full frequency analyses are shown in the supplementary material (Table S4).

### Criteria

**Fig. 2:**
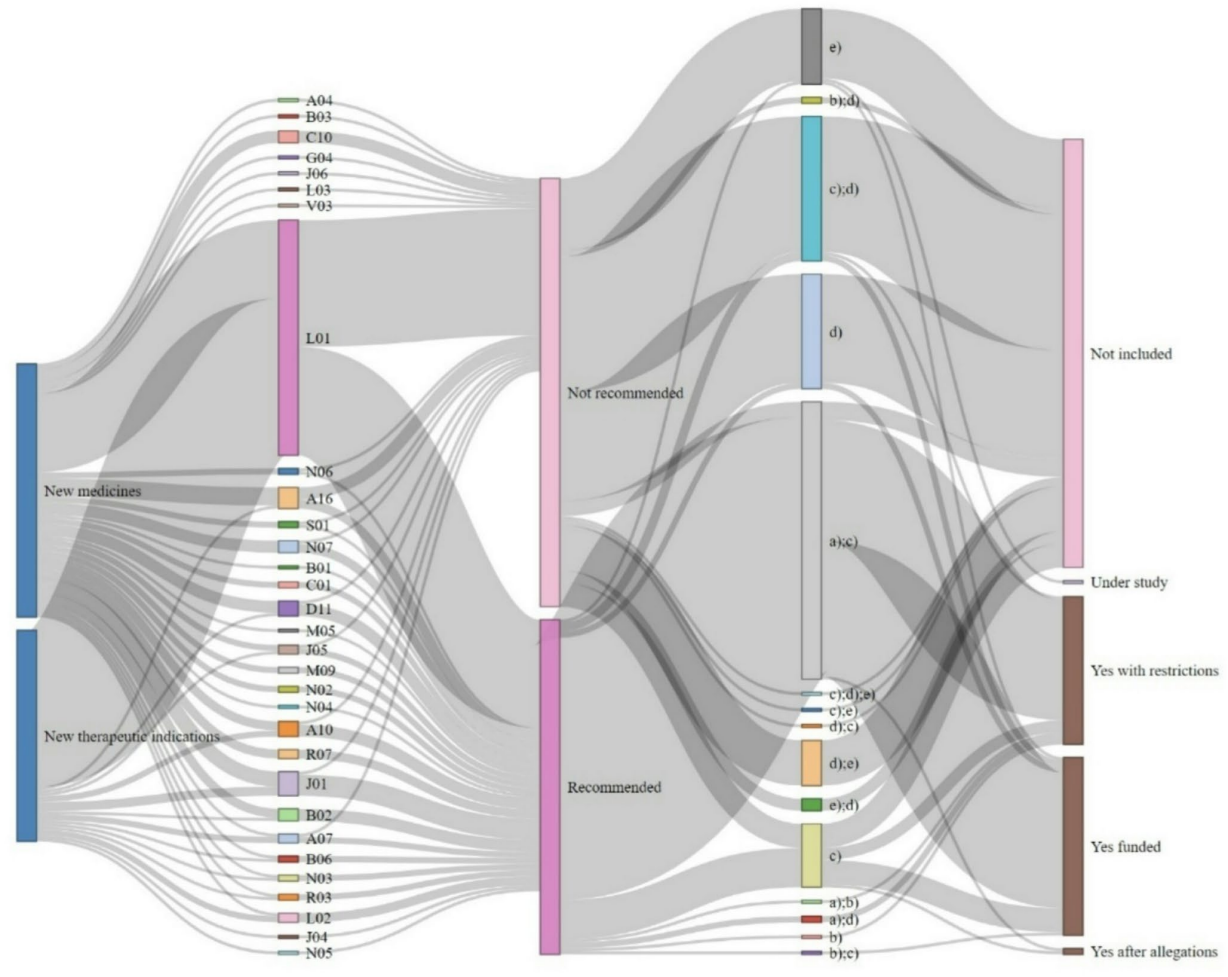
Sankey plot showing the flow of funding decisions and criteria used for reimbursement’s decisions by the CIPM in Spain between May 2019 and December 2022 Note: (**a**) Severity, duration and sequalae of the indicated conditions; (**b**) Specific needs of certain subgroups; (**c**) Social and therapeutic value of the medicines and incremental clinical benefit considering also its cost effectiveness; (**d**) Rationing of public spending on medicines and budget impact from the perspective of the SNHS; (**e**) Availability of alternative therapeutic options for the condition at the same or inferior price to that of the medicine under consideration; (**f**) Innovation

Figure 2 illustrates the flow of new medicines and new therapeutic indications through the reimbursement decision system, highlighting the key stages and outcomes of the evaluation process. The sankey plot shows two main categories, “New medicines” and “New therapeutic indications,” each represented by distinct flows. Within each of these categories, various therapeutic areas are indicated by different colour codes. As the new medicines and therapeutic indications move through the evaluation process, they are either “not recommended” or reach “recommended”. Within the recommended or not recommended categories, the plot explores the specific combinations of criteria that based the decision (e.g., a); c), b); d), c); d);e), etc.). Finally, the final reimbursement decision is categorised into several outcomes (not included, under study, yes– recommended, yes– recommended with restrictions and yes after allegations).

When combining all the positive recommendations (*n* = 110), 86 of them (78.18%) were based on criteria a) (severity) and c) (social and therapeutic value of the medicines and incremental clinical benefit considering also its cost effectiveness), and 13 (11.82%) were based on criterion c) alone. Other criteria and combination of them were used less than 4 times (see Fig. 2). The negative recommendations (*n* = 143) were mostly based on criteria c) and d) (rationing of public spending on medicines and budget impact from the perspective of the SNHS) (*n* = 46, 32.17%), criterion d) alone (*n* = 36, 25.17%), criterion e) alone (availability of alternative therapeutic options for the condition at the same or inferior price to that of the medicine under consideration) (*n* = 24, 16.78%), and criteria d) and e) (*n* = 19, 10.56%). Criterion f) (innovation) was not used to support any of the 253 decisions.

There is a moderate dependency between the decisions and the criteria (Cramér’s V = 0.4601). When looking at the individual criteria, we see that criteria a) and c) are statistically significantly associated with a positive recommendation whereas criteria d) and e) do so with negative recommendations (see Table 1).

**Table 1:** Association between recommendation and decision criteria Spain

Criterion	Reimbursement’s decisions by the CIPM		*p*-value
	Recommended	Not recommended	
a)	89 (44.06%)	6 (3.45%)	0.0000*
b)	3 (1.49%)	2 (1.15%)	0.655
c)	103 (50.99%)	62 (35.63%)	0.0000*
d)	7 (3.47%)	104 (59.77%)	0.0000*
Cramér’s V = 0.4601			

Note: p-value derived from the Fisher exact test; Percentage by column in brackets

The fact that the indication had an orphan designation or was an anti-cancer drug were not related to the recommendation, p-value = 0.673 and p-value = 0.417, respectively (see tables S5 and S6 in the supplementary material). A positive recommendation was associated with criterion c) in anti-cancer indications (p-value = 0.017, table S6).

### IPTs

Only 24 out of the 253 decisions (9.49%) mentioned the IPT in the conclusions’ section of the CIPM. All but 1 of the decisions are negative.

When looking at the relationship between the criteria and the IPTs, out of the 166 decisions based on criteria c) (alone or in combination with another criteria), only 13 (7.83%) had an IPT that mentioned economic evaluation, and just 6 of them included an economic evaluation. Only 5 included QALYs.

One hundred and eleven decisions included criterion d) as part of the rationale to support the recommendation. Only 5 out of the 111 (4.50%) corresponding IPTs mentioned a budget impact analysis and only 4 of them included it within the assessment report.

Table 2 shows the association between the actual recommendation and inferred recommendation using sentiment text analysis. While the clustering analysis provides a useful method for grouping similar IPT’s conclusions (see Figure S2 in the supplementary material), it appears that these textual similarities do not translate into predictable reimbursement outcomes. This finding suggests that the committee’s decisions are likely influenced by a variety of criteria or further evidence, many of which may not be fully captured by the textual content and sentiment of the IPT’s conclusions alone.

**Table 2:** Association between the actual recommendation and inferred recommendation using sentiment text analysis

Recommendation decision based on the sentiment text analysis	Reimbursement’s decisions by the CIPM		*p*-value
	Recommended	Not recommended	
Recommended	96 (87.27%)	127 (88.81%)	0.701
Not recommended	14 (12.73%)	16 (11.19%)	
Cramér’s V= -0.0236			

Note: p-value derived from the Fisher exact test; Percentage by column in brackets

We analysed the predictive robustness of our model by comparing its results with those obtained using an alternative sentiment test analysis that uses other dictionaries such as QDAP and Loughran-McDonald, and a different approach to the selection of relevant terms [27] Table S7 shows the adjusted odds ratio for the proportion of positive sentiment in the IPT’s conclusion associated with the final CIPM recommendation, based on logistic regression. The standardised sentiment scores are plotted in figure S3. The similarity between the two scores can be observed for most of the IPTs. The Wilcoxon rank sum test with continuity correction confirms not significant differences between the two sentiment scores.

The text analysis of the IPT conclusions revealed that only 21.74% of the IPTs included keywords aligned to the specific decision-making criteria (see table S8 in the supplementary material). This indicates that a significant proportion of the IPT conclusions do not explicitly mention the key criteria that base the reimbursement decision. For example, in 13 decisions, the IPTs mentioned the term severe or severity in the conclusion. Only 2 of these decisions included criterion a). The term subgroup does not appear in the IPT’s conclusion of any of the decisions including criterion b).

### International comparison

When conducting the international comparison, there were 87 and 36 indications whose decisions were not available at NICE and HAS’, respectively.

There does not appear to be a relationship between the Spanish and NICE’s recommendations (p-value = 0.1560, Cramér’s V = 0.1172). When grouping HAS’s recommendations based on ASMR I to III vs. ASMR IV to V, we see a positive relationship between Spain’s positive recommendations and France’s ASMR I or III, *p* = 0.001, Cramér’s V = 0.2286 (see Table 3).

**Table 3:** Relationship between Spain and NICE (England) recommendation and HAS (France) recommendation (Fisher exact test)

	**NICE**		
**Spain**	Not recommended	Recommended	*p*-value
Not recommended	14 (70.00%)	76 (52.05%)	0.156
Recommended	6 (30.00%)	70 (47.95%)	
Cramér’s V = 0.1172			
	**HAS**		
**Spain**	ASMR I - III	ASMR IV or V	p-value
Not recommended	23 (35.38%)	91 (60.26%)	0.001*
Recommended	42 (64.62%)	60 (39.74%)	
Cramér’s V = 0.2286			

Note: p-value derived from the Fisher exact test; Percentage by column in brackets

## Discussion

HTA has been generally accepted as a tool to inform evidence-based decision-making on the appropriate use of health technologies [1]. There are different ways of classifying HTA practices depending on the role, responsibility and outreach that their outputs have. For example, Fontrier, Visintin and Kanavos (2022) examined 32 countries in the EU, the UK, Canada and Australia, and produced a conceptual framework of their 63 HTA systems [2]. They found that over half of the 63 HTA systems had an advisory role, and that the majority of HTA outcomes were not legally binding. This is particularly relevant in a context where joint working between countries is being championed as a way to create efficiencies and support decision-making, particularly at the European level. Impact may be limited unless HTA outputs are indeed influencing decision-making.

Research and discussions on the need for creating synergies in HTA in the EU have been had since the mid-1990s, with the creation of the European network for HTA (EUnetHTA) in the early 2000s [28, 29]. This preceded the publication of the EU HTA 2021/2282 regulation that mandates the development of joint clinical assessments across EU member states [15]. The different EU member states have put significant effort to develop a common framework for these joint assessments, with several guidance documents and templates being developed as a result to allow an efficient collaboration. This process has not been exempted from challenges, but even so the implementation of the regulation took place in January 2025 and EU members states are working in full motion to put all the preparatory work into practice [30, 31]. However, the extent to which HTA reports actually inform final pricing and reimbursement recommendations is still unclear and subject to debate [2, 32]. Some authors advocate for a rather different approach in which the regulatory agency, usually in charge of providing a benefit-risk assessment, is the one provided with the remit of also conducting relative effectiveness assessments [33]. Arguably, this may be similar to the case of Spain for medicines, where the regulatory agency houses both the regulation and HTA of medicines. Other authors claim that unless relative effectiveness assessments have the buy-in from decision-makers in individual member states, it is unlikely they will have substantial impact in the final decision [34]. Our study somewhat agrees with this argument and highlights that unless the assessment, appraisal and decision-making process for HTA and pricing and reimbursement of medicines in Spain is aligned and based on evidence, the ongoing reform’s efforts will be futile.

Our analysis showcases that the Spanish HTA process based on the IPT had little to no relationship with the criteria used to justify medicines reimbursement’s decisions and so the impact of these reports on the final decisions has been minimal. Only about 10% of decisions on 253 indications assessed between May 2019 and December 2022 mentioned the IPT in their conclusions, and only about 22% of the IPTs’ conclusions made any reference to the criteria used to justify the reimbursement decisions. Furthermore, the criteria included in the reimbursement decisions were, on many occasions, incoherent or inconsistent with the conclusions derived in the IPT suggesting that other evidence, not in the public domain, fed into the final decision. As an example, lurasidone for the treatment of schizophrenia was originally recommended for funding based on criterion c) (social and therapeutic value of the medicines and incremental clinical benefit considering also its cost effectiveness) and on criteria a (severity) and b) (specific needs of certain subgroups) following an extension to the licensed indication, despite the IPT’s conclusion on lurasidone’s limited added therapeutic value [35–37]. This questions the relevance of these reports in the decision-making process and shows the lack of transparency in the actual criteria influencing the final decisions.

We conducted subgroup analyses for medicines with an orphan designation or those with an anti-cancer indication. Technologies for these conditions tend to be associated with special commercial agreements and therefore, one would expect this to lead to a higher association between the indication and a positive recommendation. However, we found this not to be the case. Similarly, one could suspect that conclusions for medicines with an orphan designation were to be associated with some of the criteria such as the ‘severity’ criterion. However, we found no relationship with any of the criteria in the case of orphan indications.

To our knowledge, this is the first study that includes an in-depth analysis linking the criteria influencing reimbursement decisions for medicines in Spain as specified in the legislation with the actual decisions and their relationship with HTA across all indications. At a national level, other studies have looked at the relationship between, evidence available and the reimbursement decisions for specific cases such as oncology or orphan conditions, made comparisons between HTA and pricing and reimbursement decisions between countries, or have explored the definition and application of the criteria influencing decision-making [9–12, 38–42]. At an international level, several studies have compared the HTA criteria, evidence considered, recommendations and funding decisions between European countries, many of them highlighting the need for stronger collaboration between regulatory, HTA and decision-making bodies to streamline the patient access pathway for new technologies and the need to make evaluation criteria more transparent [2, 43–46]. However, only few of these studies include Spain within analyses, particularly when looking at the final decisions [47]. A reason for this may be the difficult task of identifying the final decision and linking it to any evaluation criteria.

We conducted an international comparison between Spain decisions and those from England and France. Although this comparison cannot be understood as a like-to-like, our analysis shows that there seem to be no relationship between the Spanish Ministry of Health and NICE’s decisions in the UK. NICE’s decisions are driven by clinical and cost-effectiveness criteria alongside other aspects that are included either quantitatively in the cost-effectiveness results (such as the severity of the disease), or deliberatively (such as the level of uncertainty or health inequalities) [19]. Although Spain is meant to consider cost effectiveness as a relevant criterion in decision-making, our analysis shows that cost-effectiveness analyses have rarely been included in the HTA reports. Similarly, although the Spanish Ministry of Health has recently published a guide to the methods for economic evaluation describing a systematic approach for how these analyses are meant to be conducted [48], evidence on its implementation is still to be produced. This difference may be one of the criteria influencing the lack of similarity between the 2 countries’ outputs. Nevertheless, the current lack of a decision-making framework risks that any criteria are considered inconsistently in the decision-making process.

The ongoing Spanish reforms also suggests the potential introduction of a more systematic approach to evaluations including the introduction of a categorization of added therapeutic value, as it is currently the case in France with the ASMR scale. The international comparison in our study shows that there seems to be a positive relationship between Spain’s positive recommendations and France’s conclusion on medicines with an ASMR I or III, that is, medicines considered to have a major or moderate added clinical value. Technologies categorized with these gradings are also required to include a cost-effectiveness analysis in France. Although applying a categorization could provide a more consistent approach to assessing clinical effectiveness, implementing such a system is not free from challenges, as seen in France [49]. The draft royal decree on the evaluation of medicines in Spain does not sufficiently address the complexities involved in developing clear, mutually exclusive criteria for categorization or how societal preferences and value judgments will be factored in and the project for the draft new royal decree on the pricing and evaluation of medicines do not provide details on this aspect either, beyond mentioning that this will be an important factor to consider [3, 6].

### Strengths and limitations

Limitations of our study include the fact the analysis is limited to information available in the public domain, whilst the CIPM documents imply that other information plays a role in the final decision. The sample size of our analysis was narrowed since not all medicines had an associated IPT, and, in some cases, it was difficult to match the corresponding IPT for the specific indication. This difficulty was aggravated when the dates from the IPT and the CIPM differ substantially. The sample used for the international comparison was also limited due to differences in the evaluations available in each country. However, we believe the sample to be sufficiently large to demonstrate robust results. In fact, the exhaustiveness and completeness of the database is one of the key strengths of the study. Applying a unique identifier for evaluations and subsequent decisions would improve traceability of the medicine’s pathway throughout the evaluation and pricing and reimbursement process in Spain and should be considered during the implementation of the new process for HTA.

It is also acknowledged that in our analysis we have mainly focused on the IPT’s conclusions. This may have missed some information relevant to the criteria used for decision-making. However, the methods used to analyse this qualitative information including sentiment text analysis, keyword extraction, decision analysis, and clustering provide a systematic approach to analyse a large sample of unstructured data, adding to the existing literature when comparing funding decisions and criteria influencing those, in which analyses have mainly focused on either analysing final decisions in a large sample [50] or providing an in depth-analysis of the reasons for funding decisions with application to a very few cases [51].

### Policy considerations

The draft royal decree on the evaluation of health technologies envisages the separation of functions for the assessment, therapeutic positioning of the medicine and final decision making [3]. This structural change could address the current disconnection by ensuring that assessments directly inform appraisals. However, the absence of clear guidelines on how the appraisal stage will be conducted, and how it will integrate the assessments, leaves a significant gap in the transparency and consistency of decision-making. The project of the new royal decree for regulating the pricing and reimbursement system for medicines commits to the revision and further definition of the criteria used for final pricing and reimbursement decisions of medicines. This should be the basis of a structured decision-making framework that aligns with the HTA evaluation. The ongoing reform also signals a move toward improving the quality of HTA assessments by aligning with the EU’s joint clinical assessments under the EU HTA Regulation [30]. As highlighted in other studies [12, 13, 39, 52], unless the appraisal is clearly aligned with the evidence arising from the assessments, based on a pre-specified value framework and conducted in a transparent and deliberative manner, there is a risk that the same issues of inconsistency and lack of transparency will persist. The absence of such a framework also makes it difficult for stakeholders to understand how decisions are made, potentially undermining trust in the system [53]. The ongoing reform of the HTA and pricing and reimbursement system for medicines in Spain provides a unique opportunity to develop an evidence-informed deliberative framework that transparently describes the evidence assessed, how this feeds into the decision and the criteria and value judgements that influence the final decision, making all this available in the public domain. Our study provides a comprehensive review that can be used as a basis to develop such a framework.

## Conclusions

This study has shown that until now, the criteria used to justify the reimbursement decisions of medicines in Spain do not align with the information included in the HTA assessments. If Spain truly aspires to have a high quality, robust and transparent HTA and reimbursement process, then it must tackle this issue by developing an appraisal framework that is consistent with the assessment stage and that considers the views and input of relevant stakeholders.

While the recently published legislative and policy documents introduce promising changes aimed at enhancing the quality, transparency, and relevance of the HTA process in Spain, several key issues remain unresolved. To achieve a truly robust and transparent HTA and reimbursement system, it is essential that the ongoing reform addresses these gaps, particularly in terms of detailing the appraisal framework, ensuring meaningful stakeholder involvement, and clearly articulating how clinical, economic, and other criteria will be balanced in decision-making.

## Electronic supplementary material

Below is the link to the electronic supplementary material.

Supplementary Material 1

Supplementary Material 2

## Declarations

### JEL

I18 Government Policy • Regulation • Public Health.

The corresponding author is an employee of the National Institute for Health and Care Excellence (NICE). The work has been conducted outside of the terms of the employment.
