# Supplementary material for: Challenges and reforms in Spain’s health technology assessment system: analysis of criteria influencing medicines’ reimbursement decisions between 2019 and 2022 in Spain
Source: Eur J Health Econ. 2025 May 9;26(9):1569–79. doi: 10.1007/s10198-025-01790-7 (PMC12618429; doi:10.1007/s10198-025-01790-7)
Supplement: Supplementary file 1 — Supplementary Material 1 [file 10198_2025_1790_MOESM1_ESM.docx]

Title: Challenges and Reforms in Spain’s Health Technology Assessment System: analysis of criteria influencing medicines’ reimbursement decisions between 2019 and 2022 in Spain

European Journal oh Health Economics

Authors: Pilar Pinilla-Dominguez^1,2^, Jaime Pinilla-Dominguez^1^

^1^ Department of Quantitative methods in Economics and Management, School of Economics, University of Las Palmas de Gran Canaria (ULPGC), Las Palmas, Spain

^2^ NICE Advice, National Institute for Health and Care Excellence, London, UK

Corresponding author: Pilar Pinilla-Dominguez [maria.pinilla101@alu.ulpgc.es](mailto:maria.pinilla101@alu.ulpgc.es)

# Supplementary Material

Box S1. Decision-making criteria for public reimbursement of medicines in Spain

1. Severity, duration and sequalae of the indicated conditions
2. Specific needs of certain subgroups
3. Social and therapeutic value of the medicines and incremental clinical benefit considering also its cost effectiveness
4. Rationing of public spending on medicines and budget impact from the perspective of the SNHS
5. Availability of alternative therapeutic options for the condition at the same or inferior price to that of the medicine under consideration
6. Innovation

Source: Real Decreto Legislativo 1/2015, de 24 de julio, por el que se aprueba el texto refundido de la Ley de garantías y uso racional de los medicamentos y productos sanitarios.

Table S1: Comparison of key aspects of the HTA and pricing and reimbursement process for medicines in Spain and the new proposals within the ongoing reform, and the alignment with the EU HTA regulation

| **HTA, P&R process until 2023** | **Proposed HTA, P&R process as per January 2025** | **Alignment with EU HTA regulation 2021/2282 (January 2025)** |
| --- | --- | --- |
| HTA process based on therapeutic positioning report (IPT) not regulated in legislation | HTA process regulated in legislation as per draft Royal decree | 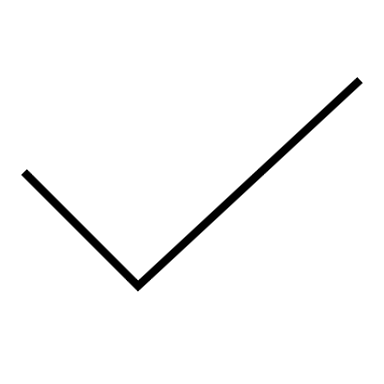 |
| HTA report developed by AEPMS with input from the autonomous regions and ad hoc patient involvement | HTA report developed by an "Office for the evaluation of the efficiency of health technologies" attached to AEPMS with systematic patient input (exact process to be defined) | HTA report developed by assesor and co-asseror from designated member states (in Spain to be designated by AEMPS) with systematic patient input |
| HTA report (IPT) did not include a recommendation | HTA report does not include a recommendation | 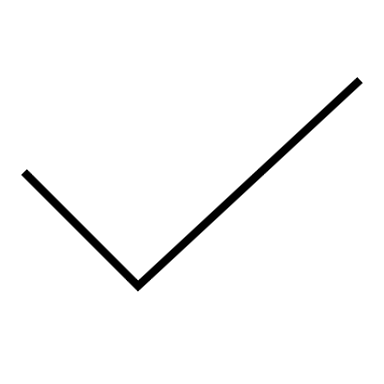 |
| HTA report (IPT) included mainly clinical aspects, with economic aspects considered in few reports | HTA report to include clinical, economic, ethical, organisational, social and legal aspects | 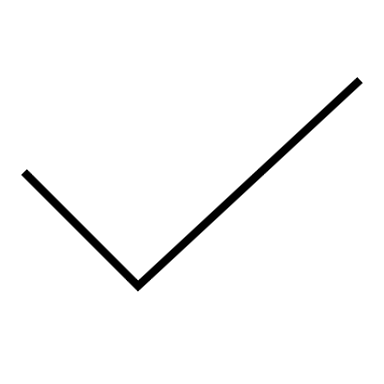 for clinical assessments |
| Therapeutic positioning decision taken by same personnel carrying out the assessment | Therapeutic positioning decision taken by "Group for the positioning of health technologies" (excludes people involved in the HTA assessment) | NA |
| Specific therapeutic positioning decision included as part of HTA report and guided by pricing and reimbursement decision | Specific therapeutic positioning recommendation separated from HTA report and pricing and reimbursement decision, and based on the appraisal of the HTA report | NA |
| HTA meant to inform pricing and reimbursement decisions as per procedural documents | Draft legislation explicitly mentions using HTA report to inform pricing and reimbursement decisions | HTA meant to inform individual member states’ decisions |
| Final reimbursement by the Spanish Ministry of Health based on the following (undefined) decision-making criteria specified in the legislation: a) severity; b) specific needs of certain subgroups; c) social and therapeutic value of the medicines and incremental clinical benefit considering also its cost effectiveness; d) rationing of public spending on medicines and budget impact from the perspective of the Spanish National Health Service; e) availability of alternative therapeutic options for the condition at the same or inferior price to that of the medicine under consideration; and f) innovation | Final reimbursement by the Spanish Ministry of Health based on revised decision-making criteria to be specified and further defined in upcoming reform. Criteria considered include additional clinical benefit, efficiency, budget impact and uncertainty. Evidence included in HTA reports should link to these criteria. | NA |

Abbreviations: HTA: health technology assessment; P&R: pricing and reimbursement, IPT: therapeutic positioning report; AEMPS: Spanish Agency for medicines and medical products;


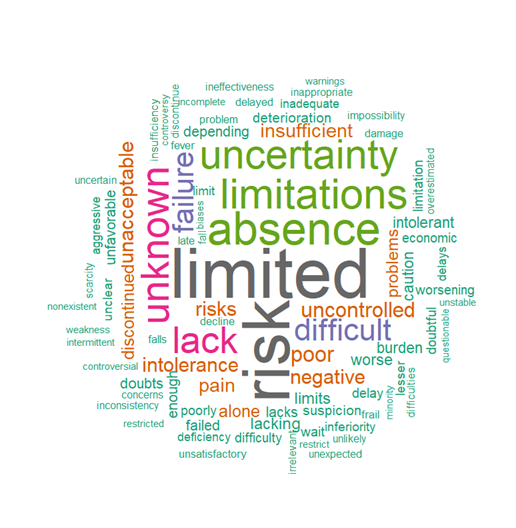
Figure S1. Word clouds of negative (left) and positive (right) sentiment keywords


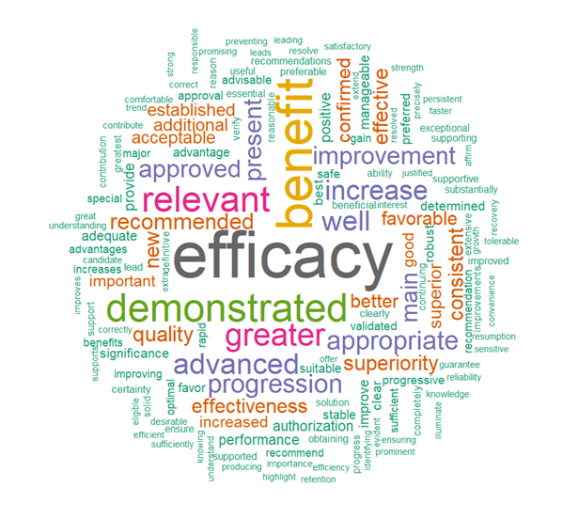


Table S2. List of words considered as negators, amplifiers, deamplifiers and adversative conjunctions

| Negators | Amplifiers | Deamplifiers | Adversative conjunctions |
| --- | --- | --- | --- |
| aint; ain't | absolutely | almost | although |
| arent; aren't | acute; acutely | barely | but |
| cannot; cant; can't | certain; certainly | faintly | despite all that |
| couldnt; couldn't | colossal; colossally | few | despite all this |
| darent; daren't | considerably | hardly | despite that |
| didnt; didn't | decidedly | incredibly | despite this |
| doesnt; doesn't | deep; deeply | kind of | however |
| dont; don't | definite; definitely | kinda | that being said |
| hadnt; hadn't | enormous; enormously | least | whereas |
| hasnt; hasn't | especially | little |  |
| havent; haven't | extreme; extremely | only |  |
| isnt; isn't | greatly | partly |  |
| mightnt; mightn't | heavy; heavily | rarely |  |
| mustnt; mustn't | high; highly | seldom |  |
| neednt; needn't | huge; hugely | slightly |  |
| neither | immense; immensely | somewhat |  |
| never | incalculable; incalculably | sort of |  |
| no; nor; not | majorly | sorta |  |
| nobody | massive; massively | sparsely |  |
| none | more | sporadically |  |
| oughtnt; oughtn't | most | very few |  |
| shant; shan't | much | very little |  |
| shouldnt; shouldn't | particular; particularly |  |  |
| wasnt; wasn't | purpose; purposely |  |  |
| werent; weren't | quite |  |  |
| wont; won't | real; really |  |  |
| wouldnt; wouldn't | serious; seriously |  |  |
|  | severe; severely |  |  |
|  | significant; significantly |  |  |
|  | sure; surely |  |  |
|  | totally |  |  |
|  | true |  |  |
|  | truly |  |  |
|  | uber |  |  |
|  | vast; vastly |  |  |
|  | very |  |  |

Note: based in Taboada, M., Brooke, J., Tofiloski, M., Voll, K., and Stede, M. (2011). Lexicon-based methods for sentiment analysis. Computational Linguistics, 37(2). 267-307

Table S3. Therapeutic indications distribution in our sample by Anatomical Therapeutic Chemical (ATC) Classification System

| ATC Second Level | Frequency | Relative frequency | Cumulative relative frequency |
| --- | --- | --- | --- |
| A03 DRUGS FOR FUNCTIONAL GASTROINTESTINAL DISORDERS | 1 | 0,40 | 0,40 |
| A04 ANTIEMETICS AND ANTINAUSEANTS | 1 | 0,40 | 0,80 |
| A07 ANTIDIARRHEALS, INTESTINAL ANTIINFLAMMATORY/ANTIINFECTIVE AGENTS | 4 | 1,58 | 2,38 |
| A10 DRUGS USED IN DIABETES | 6 | 2,37 | 4,75 |
| A16 OTHER ALIMENTARY TRACT AND METABOLISM PRODUCTS | 10 | 3,95 | 8,70 |
| B01 ANTITHROMBOTIC AGENTS | 1 | 0,40 | 9,10 |
| B02 ANTIHEMORRHAGICS | 7 | 2,77 | 11,87 |
| B03 ANTIANEMIC PREPARATIONS | 2 | 0,79 | 12,66 |
| B06 OTHER HEMATOLOGICAL AGENTS | 3 | 1,19 | 13,85 |
| C01 CARDIAC THERAPY | 2 | 0,79 | 14,64 |
| C03 DIURETICS | 1 | 0,40 | 15,04 |
| C10 LIPID MODIFYING AGENTS | 5 | 1,98 | 17,02 |
| D05 ANTIPSORIATICS | 1 | 0,40 | 17,42 |
| D10 ANTI-ACNE PREPARATIONS | 7 | 2,77 | 20,19 |
| G04 UROLOGICALS | 1 | 0,40 | 20,59 |
| H01 PITUITARY AND HYPOTHALAMIC HORMONES AND ANALOGUES | 2 | 0,79 | 21,38 |
| H02 CORTICOSTEROIDS FOR SYSTEMIC USE | 1 | 0,40 | 21,78 |
| J01 ANTIBACTERIALS FOR SYSTEMIC USE | 10 | 3,95 | 25,73 |
| J04 ANTIMYCOBACTERIALS | 1 | 0,40 | 26,13 |
| J05 ANTIVIRALS FOR SYSTEMIC USE | 7 | 2,77 | 28,90 |
| J06 IMMUNE SERA AND IMMUNOGLOBULINS | 1 | 0,40 | 29,30 |
| L01 ANTINEOPLASTIC AGENTS | 126 | 49,80 | 79,10 |
| L02 ENDOCRINE THERAPY | 6 | 2,37 | 81,47 |
| L03 IMMUNOSTIMULANTS | 2 | 0,79 | 82,26 |
| L04 IMMUNOSUPPRESSANTS | 7 | 2,77 | 85,03 |
| M05 DRUGS FOR TREATMENT OF BONE DISEASES | 4 | 1,58 | 86,61 |
| M09 OTHER DRUGS FOR DISORDERS OF THE MUSCULO-SKELETAL SYSTEM | 2 | 0,79 | 87,40 |
| N02 ANALGESICS | 3 | 1,19 | 88,59 |
| N03 ANTIEPILEPTICS | 4 | 1,58 | 90,17 |
| N04 ANTI-PARKINSON DRUGS | 1 | 0,40 | 90,57 |
| N05 PSYCHOLEPTICS | 3 | 1,19 | 91,76 |
| N06 PSYCHOANALEPTICS | 3 | 1,19 | 92,95 |
| N07 OTHER NERVOUS SYSTEM DRUGS | 4 | 1,58 | 94,53 |
| R03 DRUGS FOR OBSTRUCTIVE AIRWAY DISEASES | 3 | 1,19 | 95,72 |
| R07 OTHER RESPIRATORY SYSTEM PRODUCTS | 4 | 1,58 | 97,30 |
| S01 OPHTHALMOLOGICALS | 5 | 1,98 | 99,28 |
| V03 ALL OTHER THERAPEUTIC PRODUCTS | 2 | 0,79 | 100 |

Table S4. Distribution of indications “Recommended” and “Not recommended” and the specific combination of criteria that based the decision

| Criteria | Recommended | Not recommended |
| --- | --- | --- |
| a);b) | 1 | 0 |
| a);c) | 86 | 6 |
| a);d) | 2 | 0 |
| b) | 1 | 0 |
| b);c) | 1 | 0 |
| b);d) | 0 | 2 |
| c) | 13 | 8 |
| c);d) | 3 | 45 |
| c);d);e) | 0 | 1 |
| c);e) | 0 | 1 |
| d) | 2 | 36 |
| d);c) | 0 | 1 |
| d);e) | 0 | 15 |
| e) | 1 | 24 |
| e);d) | 0 | 4 |
| Total | 110 | 143 |

Table S5. Relationship between recommendation and orphan designation (Fisher exact test)

|  | Recommended | Not recommended | P value |
| --- | --- | --- | --- |
| Orphan: Yes | 32 | 38 | 0.673 |
| Orphan: No | 78 | 105 |  |

No relationship with individual criteria

Table S6. Relationship between recommendation and oncology indication (Fisher exact test)

|  | Recommended | Not recommended | P value |
| --- | --- | --- | --- |
| Oncology: Yes | 39 | 43 | 0.417 |
| Oncology: No | 71 | 100 |  |

Positive relationship between oncology indication and individual criterion c) (p value 0.017)

Figure S2. Principal Component Analysis conclusion plot


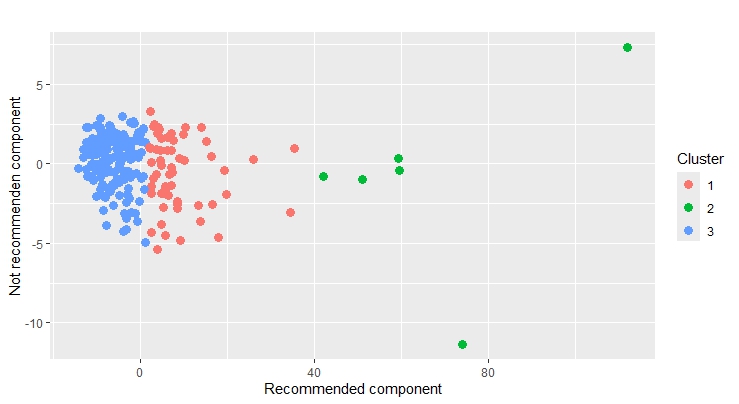


Table S7. Statistical analysis of the classifications

| Sentiment method | Odds ratio | 95% confidence interval | p -value |
| --- | --- | --- | --- |
| sentimentR | 0.8803 | 0.3817; 2.0512 | 0.8451 |
| SentimentAnalysis | 0.2513 | 0.0243; 1.4414 | 0.1433 |

Wilcoxon rank sum test with continuity correction

W = 32177, p-value = 0.9167

alternative hypothesis: the difference between the two sentiment scores is not equal to 0

Figure S3. Sentiment score distribution by Interministerial Pricing Committee of Medicines decision


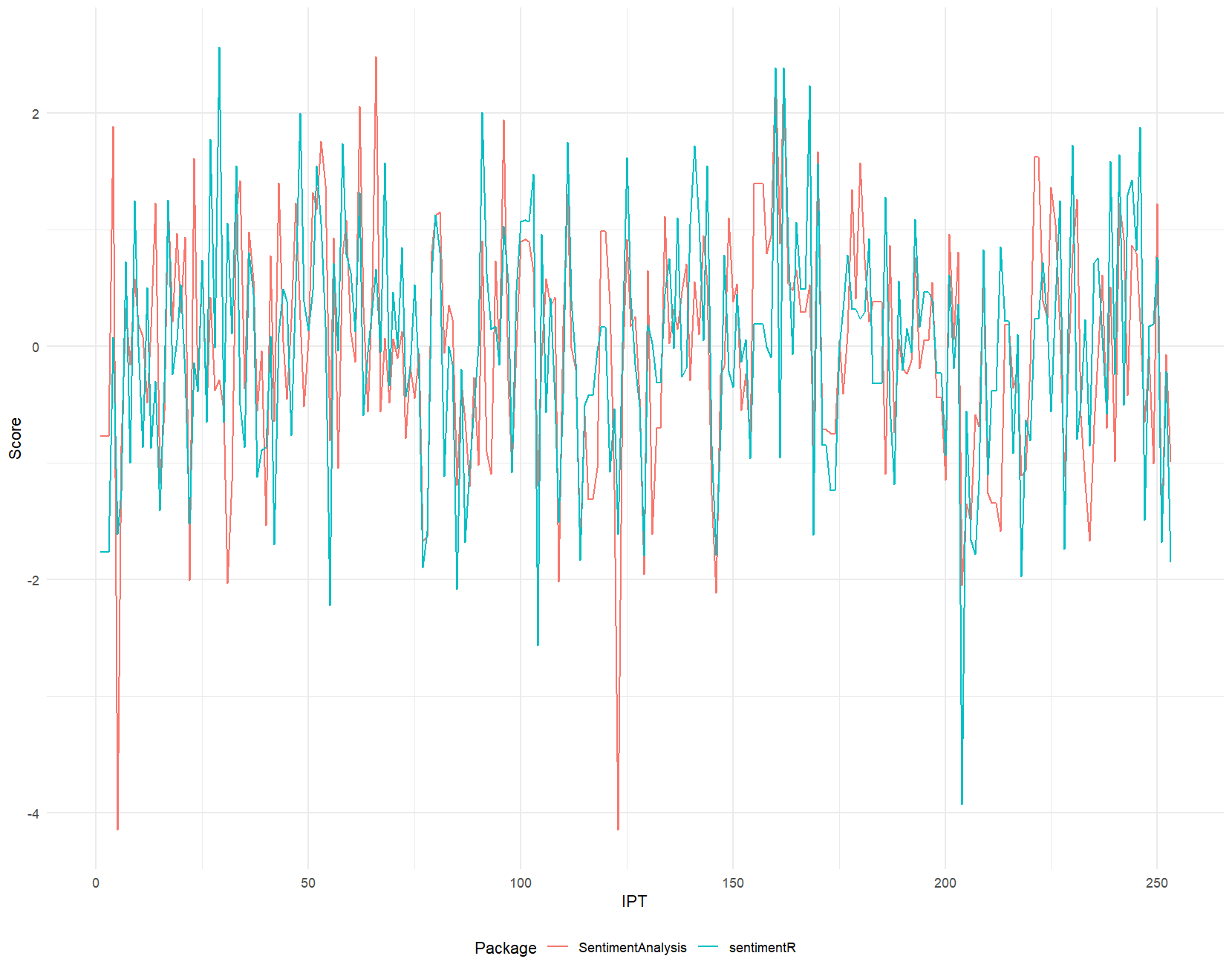


Table S8. Keywords present in the conclusions of the therapeutic positioning report aligned with the Interministerial Pricing Committee of Medicines decision criteria

| Decision-making criteria for public reimbursement | keyword | keywords present in the IPTs aligned with the decision criteria |
| --- | --- | --- |
| a) | severity | severity |
| b) | specific needs |  |
| b) | subgroups | subgroups |
| c) | social value |  |
| c) | therapeutic value | therapeutic value |
| c) | incremental clinical benefit |  |
| c) | cost effectiveness |  |
| d) | public spending |  |
| d) | budget impact | budget impact |
| e) | alternative therapeutic options |  |
| f) | innovation |  |
